# Online infidelity: The new challenge to marriages

**DOI:** 10.4103/0019-5545.58299

**Published:** 2009

**Authors:** Angelina Mao, Ahalya Raguram

**Affiliations:** Department of Mental Health and Social Psychology, National Institute of Mental Health and Neuro Sciences (NIMHANS), Bangalore, India

**Keywords:** Depression, marriage, marital therapy, online infidelity

## Abstract

Increased usage of the Internet has given rise to a new challenge to marriages: That of online infidelity, which is perceived to be as traumatic as actual infidelity. This article highlights the negative impact of online infidelity on marital relationship and its detrimental effect on the mental health of the offended spouse using a case vignette. The article discusses the importance of marital therapy in dealing with the factors contributing to online infidelity and in rebuilding marital trust.

## INTRODUCTION

Infidelity is commonly understood as a violation of the marital agreement, a betrayal of one's trust, and a threat to the marital bond. Infidelity research has addressed two types of betrayal that occur: Sexual and emotional infidelity, with online infidelity being the latest area of research. There exists a wealth of literature on the topic of online infidelity in the west, which is in sharp contrast to the lack of any published article on this issue in India. The aim of this article is to introduce the concept of online infidelity and sensitize mental health professionals to this emerging new trend. It further illustrates the occurrence of this problem in marriage using a case vignette and presents treatment strategies that were employed in working with the couple.

### Definition of online infidelity

With the development of the Internet, the definition of infidelity now includes a romantic and/or sexual relationship with someone other than the spouse, which begins with an online contact and is maintained mainly through electronic conversations that occur through e-mail and chat rooms.[1] The emphasis is on the process whereby individuals already involved in a committed relationship seek to be involved in computer synchronous, interactive contacts with members of the opposite sex. A cyber affair can either be a continuous relationship specific to one online user or a series of random erotic chat room encounters with multiple online users. There exists a debate on whether chat room contacts constitute ‘infidelity’. Mileham[2] has defined it as amounting to infidelity based on three factors: First, the institution of marriage involves emotional and sexual exclusivity and hence, sexual involvement with someone other than the spouse is considered unacceptable. Second, it typically occurs in secrecy, and is usually kept hidden from the spouse. Third, the consequential nature of chat room liaisons and the breach of trust it can create, substantiate their classification as infidelity. Most spouses feel as betrayed, angry, and hurt by online infidelity as they would if skin-to-skin adultery had taken place.[3,4] Mileham also specifies that in cases where chat room activities are not hidden from one's spouse, this definition does not apply.

## CASE REPORT

### Presenting problem

A 29-year-old married woman and home maker, diagnosed with moderate depression, presented for therapy following the discovery of her husband's chats of a sexual nature with his cyber chat partner via Internet, since the last six months. She reported that this was the second time she had caught him engaging in a sex chat with the same partner: Her husband had been friends with the cyber partner for five years. However, chats of a sexual nature had begun since the last one-and-a-half years, which she discovered for the first time eight months ago. She had become increasingly suspicious of her husband as he spent considerable time chatting on the Internet and had on occasions compared her with his chat partner, whom he had described as ‘smarter and more intelligent’. A subsequent search of the recent chat room conversations revealed that they had been sex chatting. When she confronted her husband, he denied they were having an affair, but readily agreed to discontinue chatting with his cyber partner. She was extremely hurt, but decided to put the issue behind them and move on with their lives as before. A few months later the husband again began spending long hours on his computer, late into the night. He explained that it was due to his trying to meet deadlines for some ongoing projects at work. She initially accepted his explanations as he did have a very demanding job. However, she noticed that when she approached near him while he worked on his computer he would immediately close down his computer windows. A search to confirm her suspicions proved correct again when she discovered that her husband had resumed sex-chatting two months later with the same person, who chatted with him using a different name. She was devastated with husband's ‘cheating’ on her. Her distress was heightened by her husband's strong denial of the issue as infidelity on his part.

In addition, she expressed her dissatisfaction at being a homemaker and felt inferior to her friends who had been less successful than her in school but were now doing well in their careers.

The marital evaluation suggested the possibility of other difficulties in the marital relationship and hence it was deemed necessary to explore this area in detail. The husband was invited to participate for conjoint sessions and he accompanied his wife for the following session. Although he admitted that he had been cybersex chatting, he did not perceive it as infidelity. He felt it was not so, as there had been no real physical contact and he merely engaged in it to ‘unwind and relax’ after work.

### Case formulation

Detailed exploration of the couples overall satisfaction with the quality of their marital relationship revealed that both were dissatisfied with it since the last few years. She reported being increasingly unhappy with her husband's long working hours, which became even longer after his promotion at work (husband returned home by midnight or even later, on most days). Additionally, enquiry about the level of satisfaction with their sexual relationship revealed that husband was sexually dissatisfied. The couple had been resorting to coitus interruptus as husband was allergic to latex and the wife could not tolerate the side effects of contraceptive pills. She did not report sexual dissatisfaction, however. The husband's rationalization of his action as not amounting to infidelity resulted in its continuance, despite awareness of his wife's objection and distress related to it. Differences in the couple's perception of the issue resulted in the current impasse.

Further, she had a growing sense of dissatisfaction with the trajectory of her life. She felt capable of achieving much more, but had not actively pursued her ambitions. As her life became increasingly centered around her home and husband, opportunities for developing peer relationships also considerably reduced. This situation greatly eroded her self-esteem and left her with a sense of frustration. Against this backdrop, being let down by her husband deeply hurt and disappointed her and she felt that life had become meaningless.

### Course of treatment

Marital therapy was planned in the light of the fact that the current issue was clearly embedded in the ongoing relationship difficulties. The couple was seen for eight sessions over a period of two months. Initial work focussed on helping the wife ventilate her anger and distress. The therapist ‘psycho-educated’ the husband about online infidelity,[5] regarding rationalization of his action. The therapist, using a non-confrontational stance pointed out the paradoxical manner in which he had maintained secrecy about his behavior, and at the same time regarded it as ‘not cheating’. The husband was able to acknowledge this and also reflected on the impact of cybersex on their marriage. He subsequently expressed regret over his behavior and apologized to his wife. His justification for his behavior as a way of relaxing or unwinding was also pointed out as paradoxical, as he reported experiencing physical exhaustion from spending excessive time on the computer at work, but also ended up utilizing his leisure time only on the computer. He was encouraged to exercise self-control in using the Internet during leisure time with regard to his choice of sites (for blogging, updating on information, etc.).

Keeping in mind the practical limitation of her husband's long working hours, the next stage of therapy focused on enhancing intimacy between the couple by encouraging them to identify ways by which they could spend more time together. They were able to think of joint activities that both enjoyed and taking brief vacations during weekends, as methods to enhance emotional bonding.

Subsequent to enquiry about their sexual relationship, the couple had approached their gynecologist who suggested a newer contraceptive pill, which did not produce side effects and they no longer had to engage in coitus interruptus. Wife's uneasiness about her conservative attitude towards sex was discussed and misconceptions that she held were clarified. She felt more comfortable engaging in certain sexual practices that she was unsure of earlier, resulting in greater sexual satisfaction for the couple.

After the conjoint sessions had been terminated, the wife was seen individually for four sessions to help her constructively pursue the goal of completing her post graduation. This helped to enhance her self-esteem and also ensured that she spent less time brooding about the past.

### Outcome and prognosis

At termination, the wife's depressive symptoms had reduced. She was euthymic and reported adequate sleep and appetite. The couple reported that therapy had a positive impact on their relationship. The husband had accepted responsibility for his actions and was willing to work on rebuilding trust in the relationship. A follow-up two months later revealed that therapeutic gains were maintained. The wife had stopped anti-depressants as her symptoms reduced and also because the couple had planned for a second child. They engaged in several joint activities and reported greater intimacy and improvement in their sexual relationship. The husband had reduced time spent on the Internet while at home, and the wife had started actively pursuing her academic goals and was regularly setting time aside for her studies and looking forward to completing her post graduation.

## DISCUSSION

This couple responded well to therapy over a short period of time. Factors identified in contributing to the success of therapy included, the husband's willingness to engage in therapy and his accepting responsibility for his actions and the associated negative consequences on their relationship. The couple's relationship over the years had grown increasingly distant. There was no overt conflict however. This, together with the high level of motivation in both partners to rebuild their relationship were other contributory factors to the successful outcome.

Underlying problems were present in this couple's marriage prior to the infidelity. Research carried out in this area has found that a cybersexual encounter appears to be a typical symptom of an underlying problem that has existed in the marriage before the Internet ever entered the couple's lives. Pre-existing marital problems include poor communication, sexual dissatisfaction, or boredom with the relationship.[5,6] According to the authors, although these are common troubles faced by most couples, the presence of such issues increases the risk of a cyberaffair. However, this may not always be the case, as online infidelity may occur even in the absence of any inherent problems in marriage and among ‘happily married’ couples.[2]

The husband's perception of the affair as ‘not cheating because no physical contact occurred’ is in accordance with the finding in Mileham's[2] study, wherein married individuals resorted to this form of rationalization for their actions. As therapists, it is important to highlight that although virtual — it is first and foremost a form of sexual interaction involving a live partner, which damages the exclusivity of the relationship, and is hence undesirable within the context of marriage. The therapist had to maintain a neutral, non-judgmental stance while pointing out that sex-chatting with the cyber partner was unacceptable.

Online infidelity has been identified by researchers in this field as potentially devastating to the primary relationship[5,7] and caution that it may become a major factor in deteriorating marital relations.[8] Research has so far not examined the consequences of online infidelity among clinical samples. In this case, it had precipitated an episode of moderate depression in the client, which indicates the negative consequence of online infidelity on the mental health of the injured spouse. The case illustration also demonstrates that marital therapy is an effective and valid approach in dealing with online infidelity and should be considered while planning treatment strategies.

There are no published Indian studies on this issue till date; although evidence from clinical practice shows that this is an emerging problem of concern for mental health professionals, particularly marital and family therapists. More work is needed in this area so that clinicians are better informed about the phenomenon and the appropriate methods of handling it.

## CONCLUSION

Widespread use of the Internet has gradually led to a rise in online infidelity. In couples who present with this issue, an exploration into the quality of their marital relationship and sexual satisfaction is necessary. Marital therapy is implicated in dealing with the underlying issues contributing to online infidelity and in restoring marital trust. More research is needed in the area of online infidelity because of the deleterious impact it has on marriage and the consequent impact on the mental health of the partners.
